# Transforming Growth Factor-β: An Agent of Change in the Tumor Microenvironment

**DOI:** 10.3389/fcell.2021.764727

**Published:** 2021-10-12

**Authors:** Christina H. Stuelten, Ying E. Zhang

**Affiliations:** Laboratory of Cellular and Molecular Biology, Center for Cancer Research, National Cancer Institute, Bethesda, MD, United States

**Keywords:** TGF-β, tumor-stromal crosstalk, cancer associated fibroblast (CAF), EMT—epithelial to mesenchymal transition, EndMT—endothelial to mesenchymal transition, tolerogenic differentiation, metabolic reprogramming

## Abstract

Transforming Growth Factor-β (TGF-β) is a key regulator of embryonic development, adult tissue homeostasis, and lesion repair. In tumors, TGF-β is a potent inhibitor of early stage tumorigenesis and promotes late stage tumor progression and metastasis. Here, we review the roles of TGF-β as well as components of its signaling pathways in tumorigenesis. We will discuss how a core property of TGF-β, namely its ability to change cell differentiation, leads to the transition of epithelial cells, endothelial cells and fibroblasts to a myofibroblastoid phenotype, changes differentiation and polarization of immune cells, and induces metabolic reprogramming of cells, all of which contribute to the progression of epithelial tumors.

## Introduction

Cellular communication is crucial during development, tissue maintenance and tissue repair, and miscommunication can result in loss of organismic integrity, disease and death of the organism. During tumorigenesis, cells start to proliferate uncontrollably and invade the surrounding tissues to the detriment of the organism. Although altered behavior of tumor cells is a major contributor to tumor growth, it is understood that the surrounding stroma not only tolerates but supports tumor growth. The stroma of solid tumors provides structural support and supplies nutrients to tumor cells, and when tumor cells metastasize to distant organs they might more easily grow in locations that provide suitable conditions. This seed-and-soil theory of metastatic growth was first coined by Paget (Paget, 1889). Fidler and Hart (Hart and Fidler, 1980) showed a century later that melanoma cells spread to lung or ovarian tissue but not to renal tissue independent of the primary tumor site and concluded that tumor growth indeed depends on properties of the tumor cells (seeds) and host (soil). We now understand that tumor cells affect stromal cells and vice versa, and that the crosstalk between different tumor compartments contributes to tumor progression (Bhowmick et al., 2004a,b; Kaplan et al., 2005; Stuelten et al., 2008; Van Hove et al., 2021).

Cells interact with each other and the surrounding acellular matrix by releasing and sensing regulatory molecules. One of the master regulators of tumor-stromal crosstalk is TGF-β. TGF-β instructs cell proliferation and death, cell metabolism, cell motility and migration, tissue repair, and organ development (Morikawa et al., 2016). In tumors, TGF-β acts as a tumor suppressor during early stages of tumorigenesis by inhibiting cell proliferation and promoting cell death. As tumors progress, TGF-β promotes tumor growth and metastasis by inducing a mesenchymal transition of epithelial and endothelial cells, inducing myofibroblastoid differentiation, altering differentiation and proliferation of immune cells, modulating matrix composition, and reprogramming cell metabolism (Roberts and Wakefield, 2003; Seoane and Gomis, 2017; Hua et al., 2020; Derynck et al., 2021). Through highly regulated, local activation, TGF-β has varied and context-dependent effects including the activation of specific Smad signaling cascades and alternative signaling pathways like PI3K/AKT or MAPK signaling; in addition, cross-talking with a multitude of signaling networks such as SDF1-, FGF- HGF-, EGF- or Hippo-, Wnt-, or Rho-signaling occurs (Mu et al., 2012; Luo, 2017; Zhang, 2017; Kim et al., 2018; Miyazono et al., 2018).

## Transforming Growth Factor-β Signaling: Pathways and Mechanisms

**FIGURE 1:**
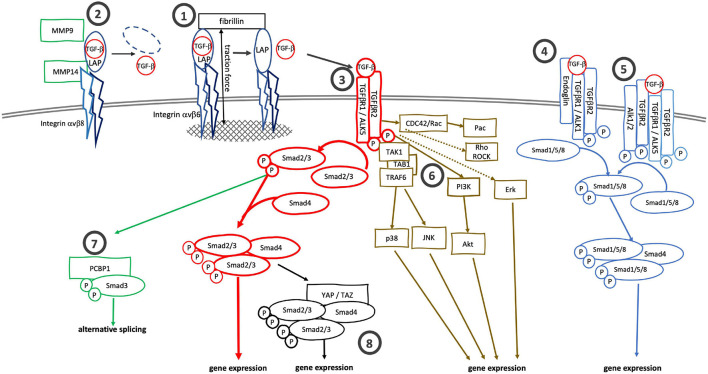
Canonical and alternative TGF-β signaling cascades. TGF-β can be activated by **(1)** traction force mediated release from the latent complex after binding to integrin αvβ6 or by **(2)** proteolysis after binding of the latent complex to integrin αvβ8. **(3)** TGF-β binds to specific receptors, TGFβR2 and TGFβR1 (ALK5), to initiate canonical Smad2/3 signaling. Alternatively, TGF-β can bind to **(4)** TGFβR2/ALK1/endoglin complexes or **(5)** TGFβR2/ALK5—TGFβR2/ALK1/2 complexes to activate Smad1/5/8 signaling. Alternatively to the Smad2/3 signaling cascade TGFβR2/ALK5 complexes can also activate MAPK-, Rho/ROCK- and cdc42/Rac/Pac signaling cascaded. pSmad3 itself can bind to **(7)** PCBP1 to support alternative mRNA splicing. **(8)** The binding of Smad2/3/Smad4 complexes to YAP/TAZ also alters gene expression profiles.

**FIGURE 2:**
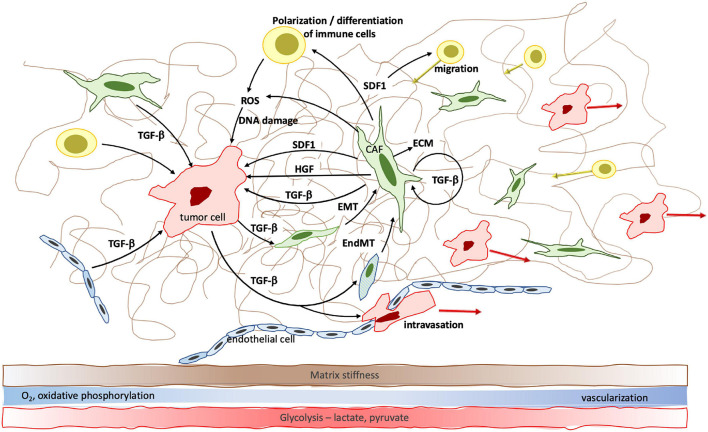
TGF-β facilitates cell-cell communication within the tumor microenvironment and changes cell differentiation, polarization and metabolism to promote tumor growth. Specifically, TGF-β induces fibroblast—myofibroblast transition, epithelial—mesenchymal transition (EMT) and endothelial—mesenchymal transition (EndMT) which result in increased cancer associated fibroblast (CAF) density. CAFs significantly contribute to increased matrix stiffness. EndMT furthermore leads to reduced endothelial cell-cell contacts which facilitates transmigration of tumor cells and metastatic spread. The effect of TGF-β on cell metabolism leads to a shift from oxidative phosphorylation to anaerobic glycolysis and accumulation of lactate, pyruvate and genome-damaging ROS in the hypoxic tumor center.

TGF-β, which exists in three isoforms, is synthesized as a propeptide consisting of the active TGF-β and the latency associated protein (LAP). The propeptide is cleaved by furin or furin-like protease during maturation, but LAP and TGF-β remain strongly associated via non-covalent interactions. LAP is tethered to latent TGF-β binding protein (LTBP) or glycoprotein-A repetitions predominant proteins (GARPs) to form latent complexes that shield the active TGF-β and prevent it from binding to receptors (Robertson and Rifkin, 2016). As such, most of the TGF-β deposited in the extracellular space is inactive, although active TGF-β is observed in specific locations (Barcellos-Hoff et al., 1994). Bioavailability of TGF-β is additionally regulated by TGF-β-binding proteins like fibromodulin and decorin which sequester TGF-β and prevent it from binding to specific TGF-β receptors (Hinz, 2015; Khan and Marshall, 2016; Nastase et al., 2018; Aubert et al., 2021). Activation of latent TGF-β is a key step in the regulation of TGF-β-signaling activity. During activation, active TGF-β is released from the latent complex by local changes in pH or shear stress, TSP1-, tenascin- or integrin binding, or by proteolytic cleavage by matrix metallo- and other proteases. Of those, integrin-mediated TGF-β activation is of particular importance, and loss of integrin-mediated TGF-β1 activation mimics the phenotype of TGF-β1-null mice (Yang et al., 2007). Likewise, mice lacking αvβ6- and αvβ8-integrins mimic the abnormalities of TGF-β1- and TGF-β3-null mice (Aluwihare et al., 2009). Integrin-mediated TGF-β activation depends on the recognition and binding of LAP’s RGD motif by integrin αv. Two mechanisms of integrin-mediated TGF-β activation are known: traction force mediated release of active TGF-β, typically seen for αvβ6 integrin (Figure 1-1), and release of TGF-β by proteolytic cleavage of LAP, observed for αvβ8 integrin (Figure 1-2; Nolte and Margadant, 2020). Integrin αvβ6 is tethered to the actomyosin cytoskeleton. After binding LAP, αvβ6 integrins link the latent complex to the actomyosin cytoskeleton. Because the latent TGF-β complex is also connected to the extracellular matrix, actomyosin generated traction forces pull on and lead to conformational changes of the latent complex and release of active TGF-β (Buscemi et al., 2011; Klingberg et al., 2014; Hinz, 2015). Notably, in this model of traction force-mediated TGF-β activation the extracellular matrix provides the counterforce for actomyosin contraction; therefore, changes in matrix stiffness should affect the traction-force mediated release of TGF-β. Indeed, integrin-mediated TGF-β activation is more efficient in stiff matrix with an elastic modulus > 10 kPa than in soft matrix (Klingberg et al., 2014; Hinz, 2015; Hiepen et al., 2020). In contrast, integrin αvβ8 does not interact with the cytoskeleton and thus cannot release active TGF-β by mechanical force transduction. It instead requires a chaperone, GARP or LRRC33, and proteases such as MT1-MMP (MMP14) to proteolytically cleave LAP and release active TGF-β (Mu et al., 2002; Liénart et al., 2018).

Once activated, TGF-β binds to TGF-β receptor type 2 (TGFβR2) to initiate signaling downstream. Upon binding TGF-β, TGFβR2 heterodimerizes with and phosphorylates TGF-β receptor type 1 (TGFβR1, ALK5) (Figure 1-3). In the canonical pathway, the activated receptor complex binds and phosphorylates receptor-regulated Smads (R-Smads), Smad2 and Smad3, which in turn heterotrimerize with the common Smad (Co-Smad), Smad4, to regulate TGF-β dependent gene expression (Shi and Massagué, 2003). The activity of the Smad signaling cascade is modulated by inhibitory Smads (I-Smad), Smad6 and Smad7, and Smurf1 and Smurf2, E3 ligases which ubiquitinylate TGF-β receptors and Smad proteins (Kavsak et al., 2000; Zhang et al., 2001; Tang et al., 2011; Nicklas and Saiz, 2013; Miyazawa and Miyazono, 2017; Yan et al., 2018; Sinha et al., 2021).

Several alternative TGF-β signaling cascades branch off the canonical signaling pathway beginning at the receptor level. Endoglin (CD105) is an accessory TGF-β receptor expressed in endothelial cells (Gougos and Letarte, 1988). Endoglin cannot bind TGF-β itself but does bind TGF-β1 and TGF-β3 when these interact with TGFβR3. Endoglin does not bind to TGF-β2 in any form (Barbara et al., 1999; Pawlak and Blobe, 2021). It facilitates the interaction of TGF-β and TGFβR2 with the non-classic type-1 receptor, ACVRL1/ALK1 (Nogués et al., 2020) and leads to a shift from TGF-β/TGFβR1/R2/Smad2/3 to TGF-β/ACVRL1/Smad1/5/8 signaling (Figure 1-4). Similarly, TGF-β can induce Smad1/5/8-signaling via formation of mixed TGFβR1/ALK5/ALK2 complexes (Ramachandran et al., 2018; Figure 1-5). In addition to Smad-signaling cascades, the activated TGFβR2/ALK5 receptor complex can activate TRAF6-TAB1-TAK1 and downstream p38 and JNK signaling (Sorrentino et al., 2008; Yamashita et al., 2008). The receptor complex can also activate PI3K/AKT signaling and feed into Ras/MEK/Erk, Rho/Rock, CDC42/Rac/Pac and Jak/Stat signaling cascades (Lee et al., 2007; Mu et al., 2012; Zhang et al., 2013; Tang L.-Y. et al., 2017; Zhang, 2017; Figure 1-6). Further downstream, activated Smad3 in the presence of CDK-, MAPK-, AKT- or PAK1-signaling can bind to PCBP1 and mediate alternative splicing (Tripathi et al., 2016; Figure 1-7).

## Transforming Growth Factor-β Signaling in Tumors

### Transforming Growth Factor-β, an Agent of Change

TGF-β is known as a potent growth inhibitor of cells of epithelial orgin, but it was first described and isolated based on its ability to transform cells and found expressed in different tumors and cell types (de Larco and Todaro, 1978; Roberts et al., 1980). In fact, TGF-β is secreted by and can act on most cells. The effects of active TGF-β are context specific (Guido et al., 2012). During development, TGF-β induces epithelial-mesenchymal transition (EMT) and facilitates gastrulation and organismic development as well as tissue repair (Thiery et al., 2009). Similarly, endothelial-mesenchymal transition (EndMT) and fibroblast-myofibroblasts transition is observed during development and tissue repair. Dysregulated EMT, EndMT and myofibroblastoid differentiation are seen in fibrotic diseases, vascular malformations, epithelial dedifferentiation and tumor growth; in advanced stages of cancers, TGF-β-induced EMT promotes tumor invasion, metastasis, and chemo-resistance (Tsubakihara and Moustakas, 2018; Katsuno and Derynck, 2021).

### Cancer-Associated Fibroblasts—Bystanders Turned Culprit

Originally considered a mere presence in tumors, CAFs are now appreciated as active partners in tumor development. CAFs can modulate stemness, proliferation, invasion and dissemination of tumor cells, ECM composition, inflammatory infiltration, angiogenesis and drug resistance. They are derived from various progenitors including resident fibroblasts, mesenchymal stem cells, adipose tissue derived stem cells, and endothelial cells. Such diverse origins confer a marked heterogeneity of CAF gene expression profiles (Calon et al., 2014; Mezawa and Orimo, 2021). Nevertheless, a core signature of TGF-β regulated ECM genes has been identified in many CAFs and goes along with poor prognosis (Navab et al., 2011; Calon et al., 2015; Chakravarthy et al., 2018).

The roles of TGF-β signaling in CAFs have been demonstrated in more detail in breast cancer models, in which TGF-β and SDF1 are part of two autocrine and cross-talking signaling loops that drive myofibroblast/CAF development at the invasive front (Kojima et al., 2010; Yu et al., 2014). Increased expression of the TGF-β target SNAI1 in fibroblasts leads to increased SDF-1 secretion (Blanco-Gómez et al., 2020). The CAF-secreted TGF-β and SDF-1 promote angiogenesis by recruiting endothelial progenitor cells, and increase growth and EMT of tumor cells (Orimo et al., 2005; Yu et al., 2014; Matsumura et al., 2019). At the same time, autocrine myofibroblast TGF-β/SDF-1 signaling attenuates expression of CD26 (Dpp4), which can cleave SDF-1, such further increasing SDF-1 signaling (Mezawa et al., 2019). Thus, once triggered, this positive feedback loop maintains myofibroblast differentiation and supports tumor progression by targeting endothelial and tumor cells.

A consequence of fibroblast-myofibroblast transition, ECM secretion by CAFs changes such that matrix stiffness and density increases. This not only impacts migration of tumor cells through the matrix, immune infiltration, vascularization and drug delivery, it also affects cell differentiation and integrin signaling. Increased ECM stiffness directly impacts epithelial differentiation via increasing integrin clustering and Erk and Rho-signaling, and promoting a malignant phenotype (Wozniak et al., 2003; Paszek et al., 2005; Lu et al., 2012). *In vivo*, the elastic modulus of tumors increases as the tumor grows, and can reach 40–50 kPa (Samani et al., 2007; Kawano et al., 2015; Wang et al., 2017), making integrin-mediated TGF-β activation more effective (Klingberg et al., 2014; Hinz, 2015; Hiepen et al., 2020) and impacting tumor progression. Indeed, high αvβ6 expression correlates with worse prognosis in breast cancer, and integrin β6 neutralizing antibody decreased tumor growth in xenograft models of breast cancer (Moore et al., 2014). On a cellular level, integrin β1 signaling is necessary for TGF-β mediated p38-signaling and EMT in mammary epithelial cells (Bhowmick et al., 2001), and in basal carcinoma, αvβ6-mediated TGF-β activation in epithelial cells leads to fibroblast-myofibroblasts transition and secretion of HGF by myofibroblasts; HGF in turn promotes invasiveness of tumor cell (Marsh et al., 2008).

Cross-talk between activated TGF-β- and YAP/TAZ-signaling can further increase matrix stiffness via alternative signaling cascades (Figure 1–8). To this end, YAP associates with Smad7 to increase its affinity to the TGFβR1 and to increase its inhibitory effect on TGF-β signaling (Ferrigno et al., 2002). Further downstream, YAP can bind Smad3 to form a YAP-TEAD4–Smad3-p300 complex on the promotor of CTGF, a cytokine involved in EMT and tumor progression (Fujii et al., 2012; Sonnylal et al., 2013; Zhu et al., 2015). TAZ controls the nucleocytoplasmic localization of the Smad2/3-Smad4 complex by binding to Smad2/3-Smad4 and increasing nuclear accumulation of Smad2/3-Smad4 (Varelas et al., 2008). In both cases, the YAP/TAZ-Smad complexes increase the fibrotic response (Piersma et al., 2015). Matrix stiffness itself can increase YAP/TAZ activation (Dupont et al., 2011) as well as TGF-β activation, forming another positive feedback circle to drive tissue fibrosis and tumor progression.

Proteolytic degradation of the ECM, for example by MMPs, is also important for tumor progression. TGF-β regulates MMP expression and MMPs proteolytically activate TGF-β. For example, tumor cell derived TGF-β can increase MMP9-secretion by fibroblasts (Stuelten et al., 2005). MMP9 in turn can bind to CD44, and then proteolytically cleave LAP and release TGF-β in addition to remodeling the extracellular matrix (Yu and Stamenkovic, 2000). As MMPs are released into the extracellular space, activation of TGF-β by this mechanism is likely less localized than traction-force dependent αvβ6-mediated activation. Other differences between these two types of TGF-β activation are that αvbβ6-mediated activation is effective in ECM stiffness, while proteolytic activation might function in soft matrix and concurs with softening of the matrix as proteins like collagens are degraded. In turn, the degradation of ECM proteins by MMPs “opens” the matrix and might allow for smoother travel of tumor cells through the extracellular space.

In summary, CAFs contribute to tumor progression by changing ECM composition and stiffness as well as the cytokine microenvironment in the tumor. As CAF-mediated changes in matrix composition spread through the environment, one might hypothesize that the resulting changes in matrix stiffness and TGF-β activation contribute to the spread of malignant cell phenotypes through the surrounding environment.

### Endothelia—More Than the Coating of the Vascular Wall

Tumors depend on blood supply for nutrients, and thus need to co-opt vessels in order to travel to distant sites. TGF-β can modulate neoangiogenesis and induce EndMT. TGF-β stimulates neoangiogenesis by inducing VEGF expression in tumor and stromal cells like macrophages in a Smad3-dependent manner (Donovan et al., 1997; Benckert et al., 2003; Kaminska et al., 2005; Sun et al., 2018). Further effects of TGF-β on endothelial cells are due the presence of the TGF-β Coreceptor Endoglin.

Endoglin has an important role in regulating angiogenesis and endothelial function (Cheifetz et al., 1992; Düwel et al., 2007; Albiñana et al., 2017). Endoglin is found to be overexpressed in the tumor neovasculature of brain, lung, breast, stomach and colon (Minhajat et al., 2006). In animal models, endoglin overexpression in tumor vasculature leads to leaky vessels with an incomplete mural coverage (Nogués et al., 2020; Ollauri-Ibáñez et al., 2020); on the other hand, haplo-insufficiency reduces the neovascularization and growth of Lewis lung tumors (Düwel et al., 2007). Mechanistically, endoglin shifts TGF-β signaling from canonical TGFβR2/ALK5-Smad2/3-signaling to the alternative TGFβR2/ALK1-Smad1/5/8 signaling cascade. While TGF-β/ALK5 signaling blocks cell proliferation, TGF-β/ALK1 signaling increases cell proliferation and motility (Lebrin et al., 2004). In addition, endoglin interacts with VEGFR2 in a VEGF-dependent manner to prevent its degradation to support tip cell formation (Tian et al., 2018). These observations support a general notion that increased endoglin expression shifts TGF-β signaling toward supporting tumor growth.

TGF-β-induced EndMT, similar to EMT, is characterized by upregulation of mesenchymal markers like α-SMA, FSP-1, vimentin and N-cadherin, by upregulation of transcription factors like Snail, Slug, Twist, and by downregulation of adhesion proteins like VE cadherin, CD31/PECAM-1 (Platel et al., 2019; Ma et al., 2020). This shift in gene expression results in endothelial cells undergoing EndMT. The loss of cell-cell contacts in the endothelial sheet during early EndMT facilitates the passing of tumor cells through the endothelial layer (Gasparics et al., 2016); later, endothelial cells acquire a pro-fibrotic phenotype with increased motility and a pro-inflammatory secretory profile, and finally convert into CAFs. Indeed, up to 40% of total CAFs in a tumor can be derived from endothelial cells (Zeisberg et al., 2007).

Mechanistically, EndMT is triggered by canonical TGF-β-signaling via ALK5/Smad2/3 or alternative signaling via TGF-β/ALK5/PI3K/Ras/TAK1 (Platel et al., 2019; Ma et al., 2020). The three TGF-β isoforms play different roles in EndMT. In colon cancer, TGF-β2 is the most important TGF-β isoform to induce EndMT (Wawro et al., 2018). Effects of TGF-β1 and TGF-β3 on EndMT are mediated by increased TGF-β2 secretion in immortalized human dermal endothelial cells, and knockdown of TGF-β2 blocks TGF-β1/2-induced EndMT (Sabbineni et al., 2018). Interestingly, the affinity of TGF-β1 and TGF-β3 to TGFβR2 is about 200–300-fold higher than that of TGF-β2 (Pawlak and Blobe, 2021). Thus, TGF-β1/3 induced ALK5 signaling might be active at low TGF-β concentrations and drive neoangiogenesis in the presence of endoglin, while TGF-β2 signaling is activate when high concentrations of TGF-β2 out-compete TGF-β1/3-binding to TGFβR2.

### Transforming Growth Factor-β and the Immune System—Suppression and Polarization

TGF-β affects the immune response to tumors on several levels: it modulates accessibility of tumors for immune cells by increasing matrix density and regulating neoangiogenesis, and it regulates proliferation, differentiation and migration of immune cells.

Generally, tumor-derived TGF-β can attract myeloid and lymphoid cells, but it also leads to immunosuppression and immune evasion of tumors by changing proliferation and differentiation of residential T cells, neutrophils and macrophages, dendritic cells and NK cells (Batlle and Massagué, 2019; Brown and Marshall, 2019). Specifically, TGF-β inhibits T-cell proliferation as well as Th1 differentiation by inhibiting IL-2 expression, and together with other cytokines promotes Treg and Th17 differentiation (Zhang, 2018). Smad3/E4BP4 signaling inhibits NK cell development and reduces immune surveillance of melanoma and lung tumors (Tang P. M.-K. et al., 2017). Furthermore, tumor derived TGF-β together with other cytokines shifts the balance of tumor associated macrophages (TAM) and neutrophils (TAN) from TAM1 toward pro-tumorigenic TAM2 (Gong et al., 2012) and from TAN1 toward pro-tumorigenic TAN2 (Fridlender et al., 2009). Together, the shift toward Treg, Th17, M2 and N2 differentiation lead to a tolerogenic immune response to tumors.

The polarization of immune cells can increase their capacity to activate TGF-β. It is worth noting that immune cells, which have high motility and are not well anchored into the extracellular matrix, often employ αvbβ8-mediated TGF-β activation which relies on proteolytic TGF-β activation, rather than αvβ6-mediated activation which relies on traction forces and requires robust cell-matrix contacts. Integrin αvb8 is found on monocytes, macrophages, dendritic cells and Tregs (Fenton et al., 2017; Nolte and Margadant, 2020). Tregs, in contrast to naïve T cells, express high levels of αvβ8 and require it to release active TGF-β from the LAP/GARP complex, which in turn leads to Treg-mediated immunosuppression (Edwards et al., 2014; Stockis et al., 2017). αvβ8-activated TGF-β is necessary to quench inflammation and auto-immunity, but also to prevent anti-tumor immunity through increased Treg activity (Brown and Marshall, 2019). Likewise, αvβ8 expression on dendritic cells leads to immunosuppression (Travis et al., 2007; Fenton et al., 2017). Furthermore, αvβ8 is upregulated on M2- and downregulated on M1-macrophages (Kelly et al., 2018). In mouse models blocking of αvβ8 by monoclonal antibodies suppresses growth of squamous cell carcinoma, mammary cancer, colon cancer and prostate cancer, emphasizing the role αvβ8/TGF-β mediated immune tolerance of tumors (Dodagatta-Marri et al., 2020).

Changes of TGF-β expression and signaling in immune cells can also contribute to tumor progression. CD2-driven overexpression of TGF-β in T lymphocytes leads to delayed tumor development in dextran sodium sulfate/azoxymethane-induced colonic tumorigenesis (Becker et al., 2004). Smad3 null mice show a variety of abnormalities of the immune system, including an activated phenotype of T-lymphocytes, impaired chemotactic response of neutrophils to TGF-β, and chronic intestinal inflammation which can concur with colon tumors in aging mice (Yang et al., 1999). Loss of Smad4 in T lymphocytes increases pro-inflammatory cytokine expression and leads to increased development of epithelial tumors (Hahn et al., 2011).

Although high TGF-β-signaling in tumors leads to immune tolerance, loss of epithelial or fibroblast TGF-β signaling increases inflammation and promotes tumorigenesis: Epithelial loss of Smad4 increases inflammatory infiltration and development of dextran-sulfate-induced colon tumors; and loss of fibroblast TGF-βRII has been associated with increased inflammation, DNA damage in epithelial cells, and tumor formation in the forestomach (Achyut et al., 2013; Means et al., 2018). Thus, dysregulation of TGF-β signaling in different tumor compartments can modulate the immune response to promote tumorigenesis.

In tumor immune microenvironment, upregulated immune checkpoints protect cancer cells from immune killing (Munn and Bronte, 2016). PD-1/PD-L1 is the currently most studied immune checkpoint pathway. TGF-β has been shown to increase PD-1 expression on immune cells, while anti-PD-1 increases tumor cell pSMAD3 and can induce immunosuppression (Baas et al., 2016; Park et al., 2016; Dodagatta-Marri et al., 2019; Wu et al., 2020). Thus, blockade of TGF-β signaling enhances the effects of PD-1 inhibitors or overcomes primary resistance to PD-1 blockade *in silico* and *in vivo* (Terabe et al., 2017; Strauss et al., 2018; Chen et al., 2021; Siewe and Friedman, 2021).

### Tumor Metabolism—A Symbiotic Relationship of Parenchymal and Mesenchymal Cells

To compensate for restricted blood and nutrient supply in tumors, another property of TGF-β comes in handy: it can shift the metabolism of cells in the tumor environment such that a symbiotic relationship between tumor cells and stromal cells results (Yoshida et al., 2019; Angioni et al., 2021).

Early on, it was observed that TGF-β increases glucose uptake and lactate secretion of cells (Inman and Colowick, 1985; Esposito et al., 1991). TGF-β signaling is now known to affect oxidative phosphorylation, the pentose phosphate pathway, glycolysis, fatty acid oxidation, and amino acid metabolism (Yadav et al., 2011; Angioni et al., 2021). In general, TGF-β shifts metabolism from mitochondrial oxidative phosphorylation toward a ketogenic metabolism, and EMT and EndMT, which are induced by TGF-β, can shift tumor and endothelial cell metabolism from oxidative phosphorylation toward anaerobic glycolysis (Angioni et al., 2021). Such a switching of the tumor metabolism from oxidative phosphorylation to anerobic glycolysis and lactate production was first described by Warburg (Warburg et al., 1927; Kim and Baek, 2021).

Mechanistically, auto- or paracrine TGF-β signaling reduces Cav-1 expression and concomitantly CD36 expression which leads to increased ROS production and HIF-1α stabilization. HIF-1α in turn increases glycolysis and increased lactate production (Guido et al., 2012; Heinzelmann et al., 2018; Yoshida et al., 2019). In tumor cells, TGF-β upregulates MCT1, increasing their capacity to uptake metabolites like lactate (Uddin et al., 2020).

The byproducts of anerobic glycolysis themselves have effects on cells and can further disturb cell and tissue physiology (Angioni et al., 2021). Specifically, lactate, which in tumors can be as high as 40 mM (Walenta et al., 2000), increases collagen production by fibroblasts and endothelial cells, endothelial cell migration and stimulates IL-8-dependent angiogenesis (Beckert et al., 2006; Végran et al., 2011). Lactate also has many effects on immune cells: it inhibits proliferation, cytokine production and cytotoxic activity of cytotoxic CD8 cells; increases ARG-1 expression in macrophages, such reducing T-cell activation and proliferation; and leads to differentiation of tolerogenic dendritic cells (Fischer et al., 2007; Nasi et al., 2013; Peter et al., 2015; Romero-Garcia et al., 2016).

In addition to its effects on energy metabolism, TGF-β-induced metabolic reprogramming of CAFs leads to increased reactive oxygen species (ROS) production and ROS accumulation by inactivation of CSK3 and the mitochondrial complex IV (Byun et al., 2012). The increased ROS levels in the tumor increase inflammation and DNA damage in tumor cells, and such further advance tumor progression.

TGF-β-mediated metabolic reprogramming of CAFs can spread to neighboring cells (Guido et al., 2012). Conceivably, once triggered, large parts of the tumor stroma might convert to a “Warburg-like” cancer metabolism. This metabolic flexibility would allow CAFs and other cells to better adapt to the changing demands of the tumor microenvironment to hypoxic and aerobic zones: in the fibrotic and hypoxic tumor core, tumor cells, fibroblasts and endothelial cells can utilize glucose by anaerobic glycolysis and secrete lactate and pyruvate, while at the oxygen-rich edges of the tumor lactate and pyruvate can be taken up by tumor cells, fibroblasts and endothelial cells and entered into the citrate cycle. In summary, TGF-β induces a metabolic plasticity that allows cells to successfully adapt to and thrive in the challenging and ever-changing tumor environment.

## Conclusion

From its discovery 40 years ago to today, TGF-β has proven to be a major player in cell biology. The tightly regulated temporospatial activation of TGF-β as well as its wide network of canonical and alternative signaling cascades and cross-talking with other signaling networks lead to cell- and compartment specific effects. Aside from the suppression of tumor cell proliferation during the early phases of tumorigenesis, the effects of the universally present TGF-β on cells are many; in their core, they relate to cell metabolism and differentiation (Figure 2). It is these effects that explain TGF-β’s unique and multifaceted role in tumor progression, from stiffening of the tumor matrix, to neoangiogenesis, to immune tolerance, and to metabolic changes throughout the varying tumor areas. As a consequence, tumor and other cells acquire increased adaptability that enables them to thrive in hypoxic, nutrient poor and stiff tumor areas as well as in the more pliable, well vascularized marginal areas, and to contribute to tumor progression.

While the mechanisms by which TGF-β exerts its functions are increasingly unraveled, many questions still remain. How are some of the effects of TGF-β compartment specific when cells are exposed to TGF-β from different sources, that is, when fibroblasts respond to tumor cell derived TGF-β but not their own, how do they sense the difference? And regarding the activation of TGF-β one wonders: Does integrin-binding of LAP merely serve the release of active TGF-β, or also lead to active integrin signaling? Does LAP have additional functions once TGF-β is released?

As research into the mechanism of TGF-β signaling is ongoing, several clinical studies exploring the effect of modifying TGF-β signaling on tumor growth have been launched in the past two decades, starting with the pan-TGF-β binding antibody ID11. Other strategies employed in modifying TGF-β signaling for therapeutic purposes include antisense oligonucleotides, small molecule receptor kinase inhibitors, and peptide aptamers (Xie et al., 2018; Liu et al., 2021). With targeting immune checkpoints as a major focus of current cancer therapies, several clinical trials with combined inhibition of PD1/PD-L1 and TGF-β are ungoing. In addition, bifunctional fusion proteins targeting PD-L1 or CTLA-4 and the TGFβR2 to inhibit TGF-β pathway and immune checkpoint simultaneously, were shown to be superior to PD-1 or CTLA-4 inhibitors in controlling tumor growth *in vitro* and *in vivo* (David et al., 2017; Lan et al., 2018; Ravi et al., 2018).

Future clarification of the cell- and context specific effects of TGF-β will help to further harness its signaling network for tumor therapy.

## Author Contributions

CS and YZ conceived and wrote the manuscript. Both authors contributed to the article and approved the submitted version.

## Conflict of Interest

The authors declare that the research was conducted in the absence of any commercial or financial relationships that could be construed as a potential conflict of interest.

## Publisher’s Note

All claims expressed in this article are solely those of the authors and do not necessarily represent those of their affiliated organizations, or those of the publisher, the editors and the reviewers. Any product that may be evaluated in this article, or claim that may be made by its manufacturer, is not guaranteed or endorsed by the publisher.
